# Supplementary figures and images for: A hybrid of light-field and light-sheet imaging to study myocardial function and intracardiac blood flow during zebrafish development
Source: PLoS Comput Biol. 2021 Jul 6;17(7):e1009175. doi: 10.1371/journal.pcbi.1009175 (PMC8284633; doi:10.1371/journal.pcbi.1009175)

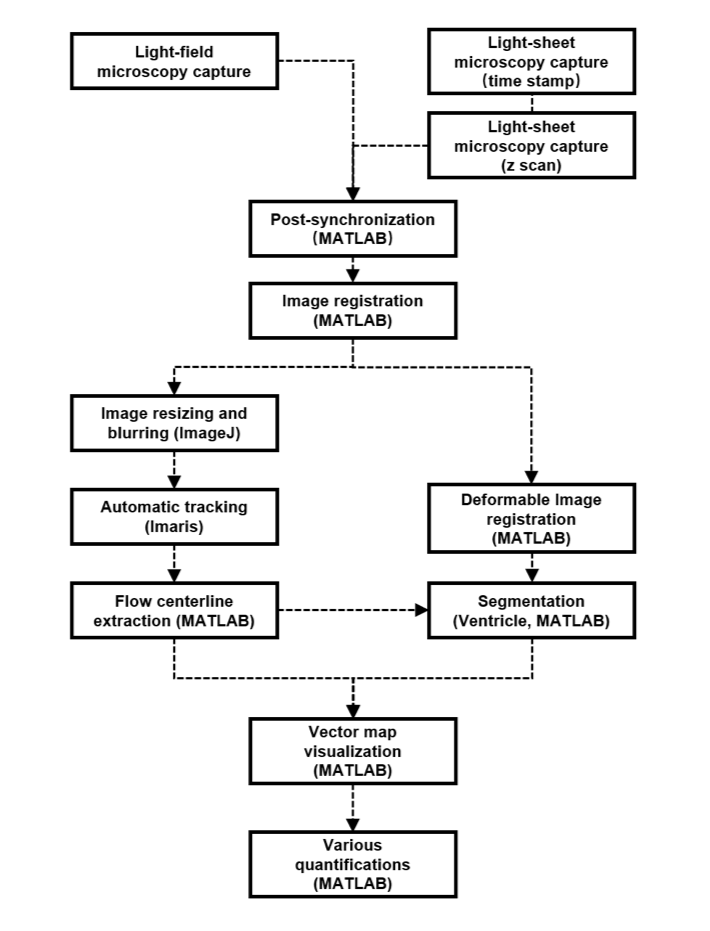

Supplement: S1 Fig — The integration of light-field and light-sheet microscopy captures and synchronizes the blood flow and myocardial motion. Images of blood cells have been re-sampled to have 3-D isotropic spatial resolution, followed by Particle Tracking Velocimetry (PTV) to map the flow velocimetry. In parallel, deformable image registration (DIR) allows for displacement analysis of the myocardium. The flow centerline, inferred from blood cells distribution, also indicates the geometry of the ventricle; thereby, facilitating segmentation of the ventricular area for displacement analysis. The dual channel data are merged and visualized at the end. (TIF) [file pcbi.1009175.s001.tif]

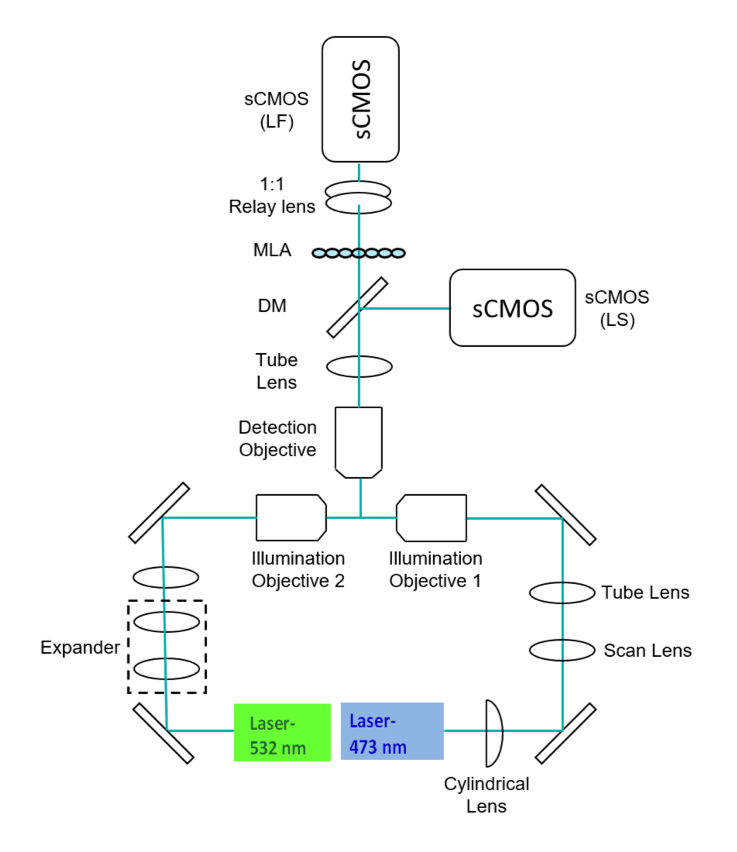

Supplement: S2 Fig — Schematic of the hybrid system. The laser with different wavelengths (473 nm and 532 nm) forms two pathways entering the opposing illumination objectives 1 & 2. Aperture, lens pairs and cylindrical lens are used to modulate the dimension and shape of the beam to form 1) the selective plane illumination for light-sheet microscopy and 2) the selective volume illumination for light-field microscopy. The fluorescent signal is collected by the detection lens orthogonal to the illumination. A dichromatic mirror (DM) partitions the detected fluorescent signal onto two detection modalities. For light-field detection, a microlens array (MLA) is placed on the intermediate image plane, and the sCMOS camera is conjugated to the back focal plane of MLA through a 1:1 relay lens. (TIF) [file pcbi.1009175.s002.tif]

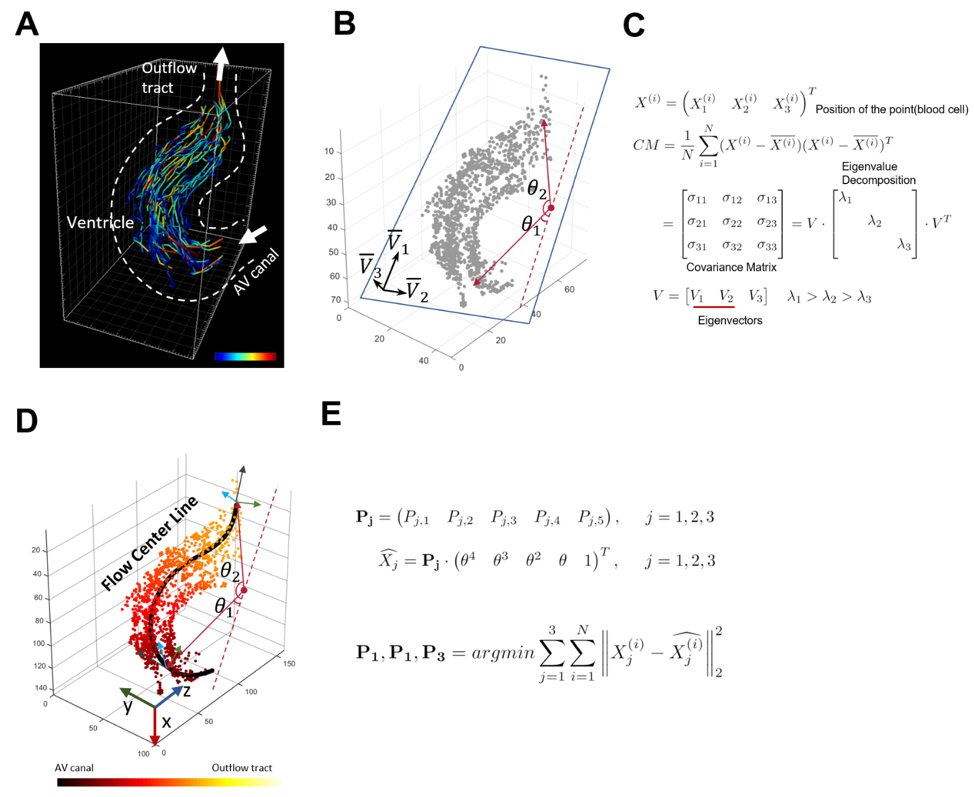

Supplement: S3 Fig — (A) Trajectories of the individual blood cells are positioned by automatic tracking throughout the cardiac cycle. (B) Principal Component Analysis (PCA) is used to define the main plane of the cell distribution (grey). The angle of each cell in reference to the center point (red) is defined as θ. (C) For the PCA analysis, the covariance matrix is computed on the 3-D coordinates. By eigen value decomposition, we extract eigen vectors of the first two principal components to define the main plane. (D) A centerline is fitted through the blood cells. For each point along the centerline, we define a new coordinate system (see axis on the line). (E) Centerline fitting is derived as a least square problem. Each coordinate (x, y or z) of the point X is fitted as the fourth order polynomial function with an angle θ. Coefficients P1, P2, P3 are computed by solving the minimization problem. (TIF) [file pcbi.1009175.s003.tif]

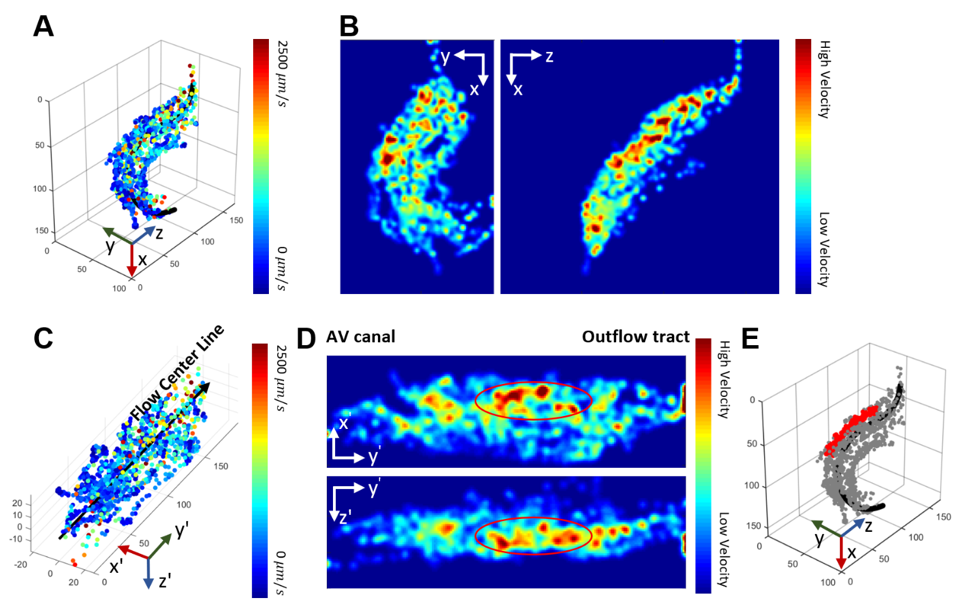

Supplement: S4 Fig — (A) Mapping of the intracardiac blood cells with color-coded magnitude reveals the distribution of average velocity during the entire cardiac cycle. (B) Heatmaps are illustrated in the projected views. (C & D) The traveling cells are visualized in the transformed coordinate system with respect to the center flow line. The swapped coordinate system provides a standard perspective to analyze the intracardiac flow dynamics, bypassing the variations from the different imaging orientations. It also defines the positions of myocardial segments (Figs 3 and 4). (E) The elevated intracardiac flow velocity is highlighted in red, corresponding to the circled regions in (D). (TIF) [file pcbi.1009175.s004.tif]

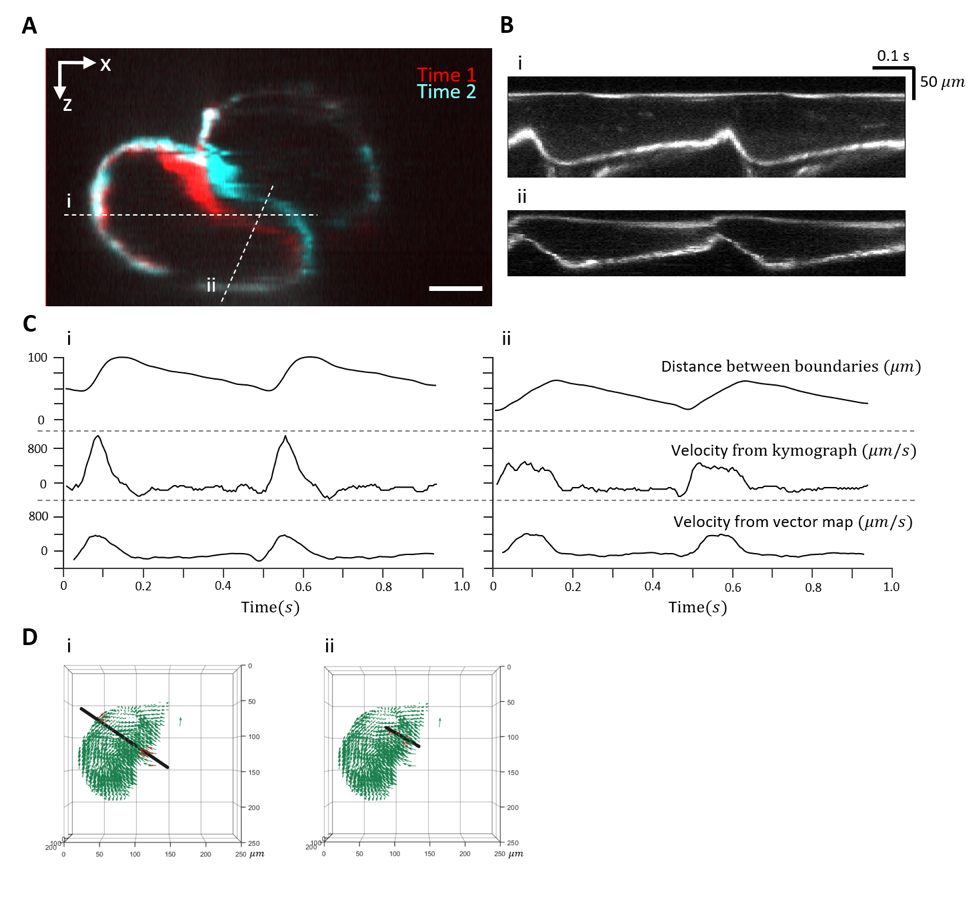

Supplement: S5 Fig — (A) x-z cross-section of hearts at two time points. Dotted lines denote the sampling positions for kymographs. Scale bar, 30 μm. (B) Kymographs display the relative displacement of the ventricle throughout the cardiac cycles. (C) Distance between boundaries are computed from kymographs. The derivative indicates the velocity of relative displacement, i.e. deformation rate. By sampling the heart in a direction (i) tilted from deformation direction (ii), one can enlarge the deformation rate drastically. Thus, kymograph based-analysis by 1D sampling is prone to angle-dependent variance. By sampling the vector map (D), a relative velocity can also be computed. In specific, the velocity is sampled at the intersection of the sampling line and the wall boundary. The relative velocity between two opposite intersection points represents the rate of relative displacement between two boundaries. It correlates with the result from properly sampled kymograph (ii) in term of magnitude and temporal pattern. (TIF) [file pcbi.1009175.s005.tif]

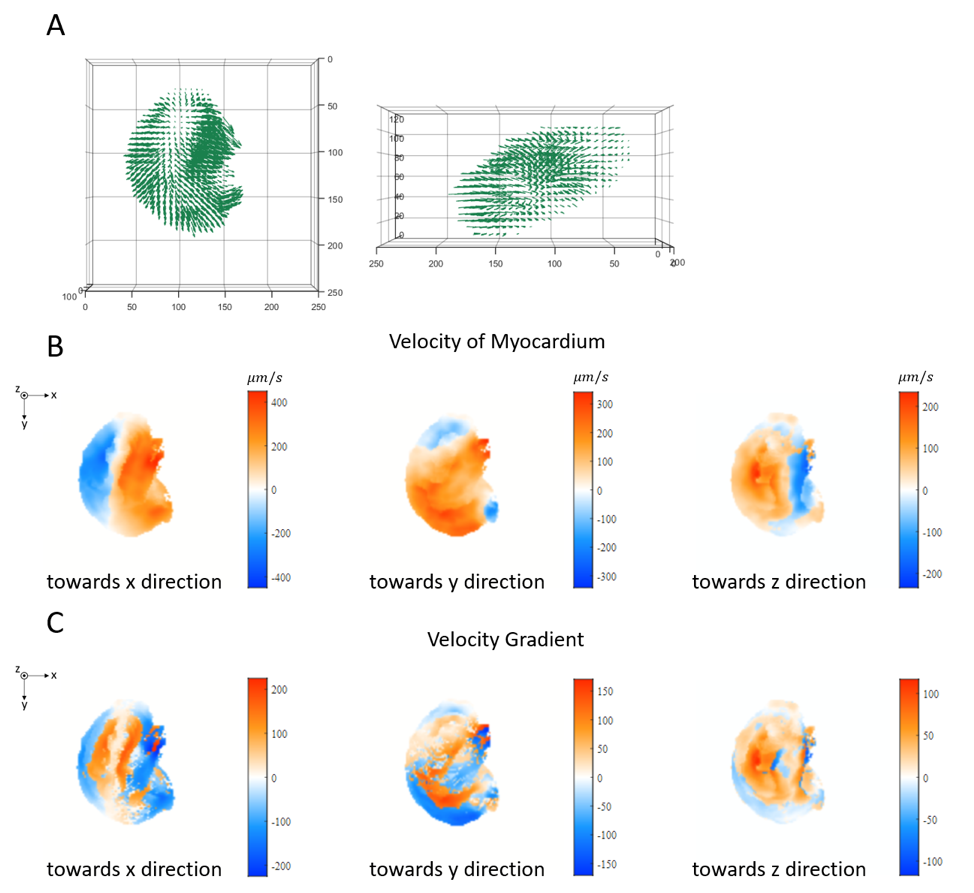

Supplement: S6 Fig — (A) The velocity field illustrates ventricular contraction from the x-y and y-z views of the ventricle. (B) The velocity of ventricular contraction is depicted in the x, y and z axis direction. (C) The gradient of velocity in the x, y and z axis reveals the differential rates of regional/segmental wall deformation. (TIF) [file pcbi.1009175.s006.tif]

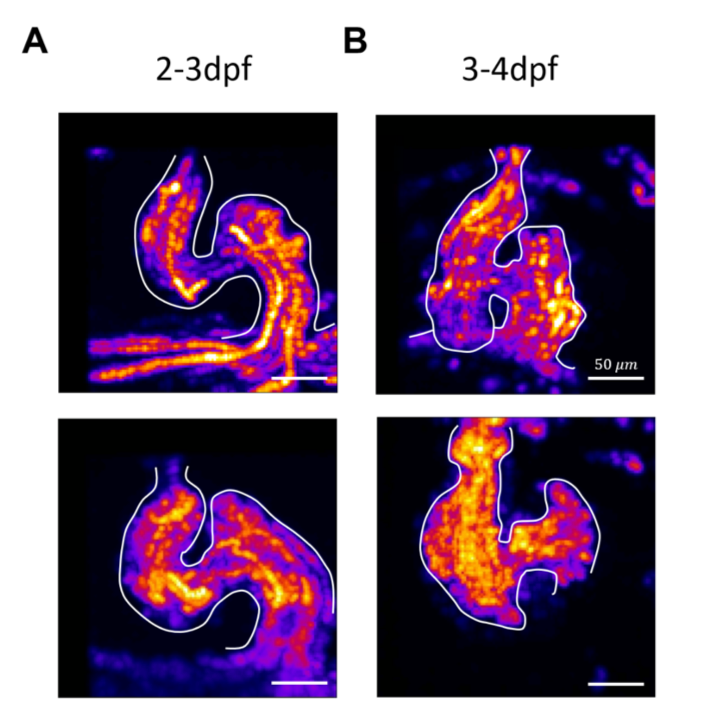

Supplement: S7 Fig — Each figure was generated by the summation of different time points over the entire cardiac cycle. (A) Data were acquired during the early stage of cardiac looping (2–3 dpf) and during the (B) late stage (3–4 dpf). (TIF) [file pcbi.1009175.s007.tif]
