# Supplementary material for: A hybrid of light-field and light-sheet imaging to study myocardial function and intracardiac blood flow during zebrafish development
Source: PLoS Comput Biol. 2021 Jul 6;17(7):e1009175. doi: 10.1371/journal.pcbi.1009175 (PMC8284633; doi:10.1371/journal.pcbi.1009175)
Supplement: Attachment — Submitted filename: Review_Wang_et_al_2020_PLOSCOMPBIOL.pdf [file pcbi.1009175.s012.pdf]

Wang et al. presented a paper about an image analysis pipeline to integrate light field microscopy (LFM) and light sheet fluorescence microscopy (LSFM) and applied it to blood flow in the heart of developing zebrafish embryos.

Main comment:
It appears hard to exactly capture the achievements that the authors want to highlight in this manuscript. If the author's particular optical integration of LFM and LSFM (i.e. the microscope) has been described in this form for the first time, then the manuscript should highlight that achievement. Currently the microscope is rather taken as a given (author's reference 20-22) and described in detail elsewhere (author's reference 22).
Similarly, the postprocessing of LFM images is complicated but seems to have been published and highlighted elsewhere (author's reference 22). The reviewer acknowledges that author's reference #22 is a preprint, and that the current manuscript's Fig 1 almost identically appears as Fig 3 in the 2019 bioRxiv version of #22. But the 2020 version of #22 uses different figures and is clearly focused on microscopy and deep learning data processing. So the reviewer assumes that microscopy and deep learning are not part of the current manuscript under review.

If we assume that the paper starts with LFM and LSFM images and aims to highlight the achievement of creating a data processing pipeline, then it is similarly hard to exactly pinpoint the author's scientific achievement highlighted in the manuscript.
It seems that the deformable image registration (DIR) used to perform vector analysis stems from the standard MATLAB toolbox and similarly, it seems that the particle tracking velocimetry (PTV) analysis is done using standard Imaris functions (see Methods 'Computation of Vector Mappings').
The authors refer to a github repository https://github.com/aaronzq/cardiac but at the time of this review, the reviewer received an '404 webpage not available' error (screenshot below). Similarly, looking through aaronzq's 18 public github repositories, no 'cardiac' repository could be found. Aaronzq's vcdlfm repo comes with a nice jupyter notebook tutorial but seems to be unrelated to this manuscript. So unfortunately, while code in the github repository could have clarified some of the questions above as to what the author's contributions to the field are, it was unfortunately not available for this review.

From personal experience, the reviewer knows how hard it is to create an end-to-end imaging system plus image processing pipeline. If this is what the authors did, and in addition creating a working application on the developing zebrafish heart, then that is fantastic. However the way the manuscript is written, it is unclear what the main achievement is and what should be highlighted.

In general, the manuscript would benefit from a clear purpose:
E.g. if:

Computational question:

- No comparison between an old tool and a new tool (PIV vs PTV is indicated in discussion but just one sentence)

Engineering question:

- Is it hard to do LFSM and LFM together? What is the prior art here?

Biological question:

- No comparison to mutants (disease)
- No comparison to different times dpf (development)

Combined Engineering, computational and biological question:
- What is the prior art? Could this never be imaged before?
- And if so, what are the surprising insights you learned, now that you see it?

In general, the manuscript would benefit a lot from a deeper description of the prior art (literature review), the aim to improve on this prior art, the obstacles along the way of implementing these improvements and the eventual result from that effort.

The reviewer thinks that the manuscript has a lot of potential. Combining LSFM and LFM is fantastic (but is it the aim of this paper?). The deep learning to process LFM is important (is it the aim of this paper?). The generation of a data processing pipeline to go from raw zebrafish heart LSFM and LFM imaging data to processed insights is important (is that the aim of this paper? How much of the work is from the authors vs the use of standard library functions of MATLAB and Imaris? An accessible github repository would help to clarify that question). The insight gained from imaging zebrafish hearts at that speed volumetrically is intriguing! (is that the aim of the paper? Where is the comparison to prior art, what is the new data? What are the surprising findings?). And if the aim is more biological: Where is the comparison to other developmental timepoints? Does the bloodflow heart dynamics change? How does the data change when mutations or other altered conditions are applied?

The reviewer recommends 1) a major revision and 2) recommends that the authors rewrite the manuscript to clarify the above questions and 3) make their github repo available to support their claims.

Minor comments:

- The authors talk about 200 fps and 200 vps. That might be confusing for some readers. The exact difference should be stated.
- Similarly: The exact difference between LFM and LSFM needs clarification. Two example sentences from the author's manuscript: "The LSFM system was capable of rapidly visualizing the embryonic zebrafish heart at 200 frames per second", "For this reason, light-field requires merely a single exposure for fast volumetric (3-D) detection up to 200 frames per seconds [23]" So are they the same speed? Why put them together then?
- Similarly, the author's sentence "Owing to the limited 3-D imaging speed associated with the current optical microscopy, uncoupling myocardial contraction from intracardiac flow dynamics to investigate ventricular structure and function remains an imaging challenge" does not specify what these limitations are (missing prior art review) and why specifically potentially combining two modalities could solve those challenges.
- Fig. 2D, the range 'low to high' would benefit from having physical units

Typos:

- The manuscript would benefit from a spell checker-run before re-submission
- "DIR with PTV techqniues "
- "uncovers sptatial"
- "flow dynamics to study to cardiac morphogenesis"

github.com/aaronzq/cardiac

Why GitHub? Team Enterprise Explore Marketplace Pricing Search / Sign in Sign up

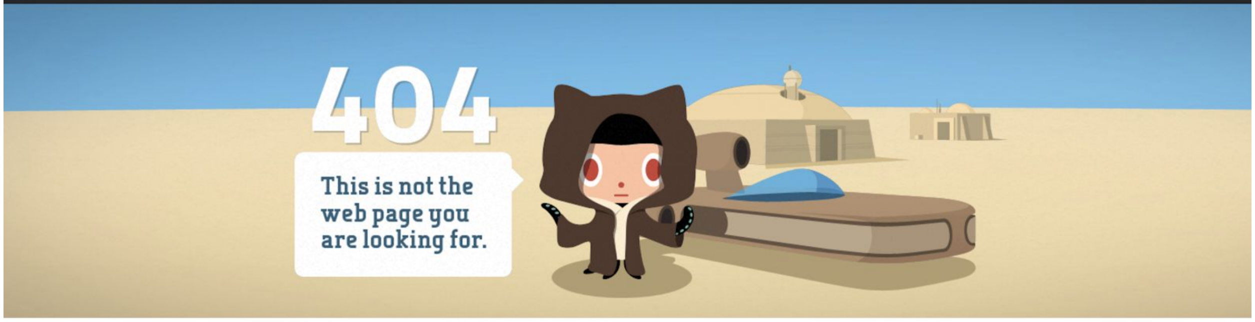

Find code, projects, and people on GitHub:

Search

Contact Support — GitHub Status — @githubstatus
