# Supplementary material for: A hybrid of light-field and light-sheet imaging to study myocardial function and intracardiac blood flow during zebrafish development
Source: PLoS Comput Biol. 2021 Jul 6;17(7):e1009175. doi: 10.1371/journal.pcbi.1009175 (PMC8284633; doi:10.1371/journal.pcbi.1009175)
Supplement: Attachment — Submitted filename: FINAL_PlosCompBio_response_letter_20210309.pdf [file pcbi.1009175.s013.pdf]

Reviewer #1: Overall, this is a really interesting and important method to separate and measure the myocardial and hemodynamic kinematics of embryonic heart contractions, something that has typically been explored computationally or at a gross functional level. Understanding how contractility initiation and propagation corresponds with (and drives) local morphogenetic events is very important for understanding mechanisms of maturation and malformation. Although this current paper doesn't address either of those topics, the presented technique sets the stage for such questions to be answered in the zebrafish model to the degree that model system can recapitulate those events.

This being said, the current contributed data does not fully reflect the capability or potential of the measurement technique. The following concerns should be addressed to improve the validation and impact of the contribution:

1) The measurement approach appears to iterate through individual planar slabs of the myocardium. While this can create cubic voxels, it is unclear how accurate/precise the in-plane motions vs. out-of-plane deformations/velocities are within a specimen. Are these known? One simple way would be to re-analyze the same specimen by rotating either the scanning field or the specimen 90 degrees. Obtaining largely identical datasets would establish how locally variable that data is and whether it is biased to the plane of inquiry.

We appreciate the reviewer's comment. For acquiring myocardial motion, our approach is to sample a 3D temporally periodic signal by a sequence of 2D slices. Despite the fact that the myocardial motion is in 3D, the signal remains periodic in each 2D pixel. Thus, we were able to, in theory, reconstruct the signals by correctly sampling each 2D pixel. To clarify this reconstruction, we could imagine that the tissue moving out of the current plane would appear in the adjacent planes. If we capture all adjacent planes, then the out-of-plane motion would still be recorded.

This approach is adapted from the previously established and validated "retrospective gating method" (Mickoleit *et al.*, 2014; Liebling *et al.*, 2005). While the specific implementation varied, these works all assumed periodicity in the heartbeat, and they used image similarity to find matched cardiac phases from originally asynchronous sequences. The results derived from this method have been successfully adopted to investigate the endocardial trabeculation (Lee *et al.*, 2016), to provide geometry for computational fluid dynamics (CFD) (Vedula *et al.*, 2017), and to map the calcium flux in the cardiomyocytes in the heart (Weber *et al.*, 2017).

Since Mickoleit *et al.* (2014) reported the mistakes with synchronization when the "heart contraction wave" propagates (from atrium to ventricle) nearly along the imaging scanning direction, it is ideal to optimize the fish orientation prior to imaging. Similarly, the orientation of the embryo is also important for obtaining a clear optical window with minimized tissue scattering (from the yolk sac, spine, etc.). Considering these, we imaged the ventral side of the fish (abdomen) in our experiments; that is, the abdomen of the fish facing up to the objective, and the atrium and ventricle are laterally positioned.

In conclusion, we think our approach provides sufficiently accurate reconstruction of the 3D motion, according to the published works. We intend to unify the orientation of the fish/heart prior to imaging. Therefore, all samples were analyzed from the same angle. However, small variations in orientation due to inter-observer variation were

inevitable among experiments. We addressed this variation by assessing several fish under the same experimental condition, as supported by our response to the Reviewer's Comment #2.

2) Related to #1, it is unclear how the local features measured in either tissue kinematics/velocities are shared in embryos of the same stage or across stages. This paper appears to measure the phenomena in an individual specimen, but does not compare across specimens for consistency across time/space. Shared features across embryos are more likely meaningful, as is an understanding of the variation present locally between embryos.

We thank the reviewer's comment. To show variations and shared features among different fish of the same days post fertilization (dpf), we repeated our experiment to gather data from 7 fish at 3 dpf. The velocities of both blood flow and myocardial displacement were measured using our processing pipeline. We sampled one cardiac cycle from each fish, normalized the time to the duration of one heartbeat, and we compared the dynamics in different fish hearts (see new figure below).

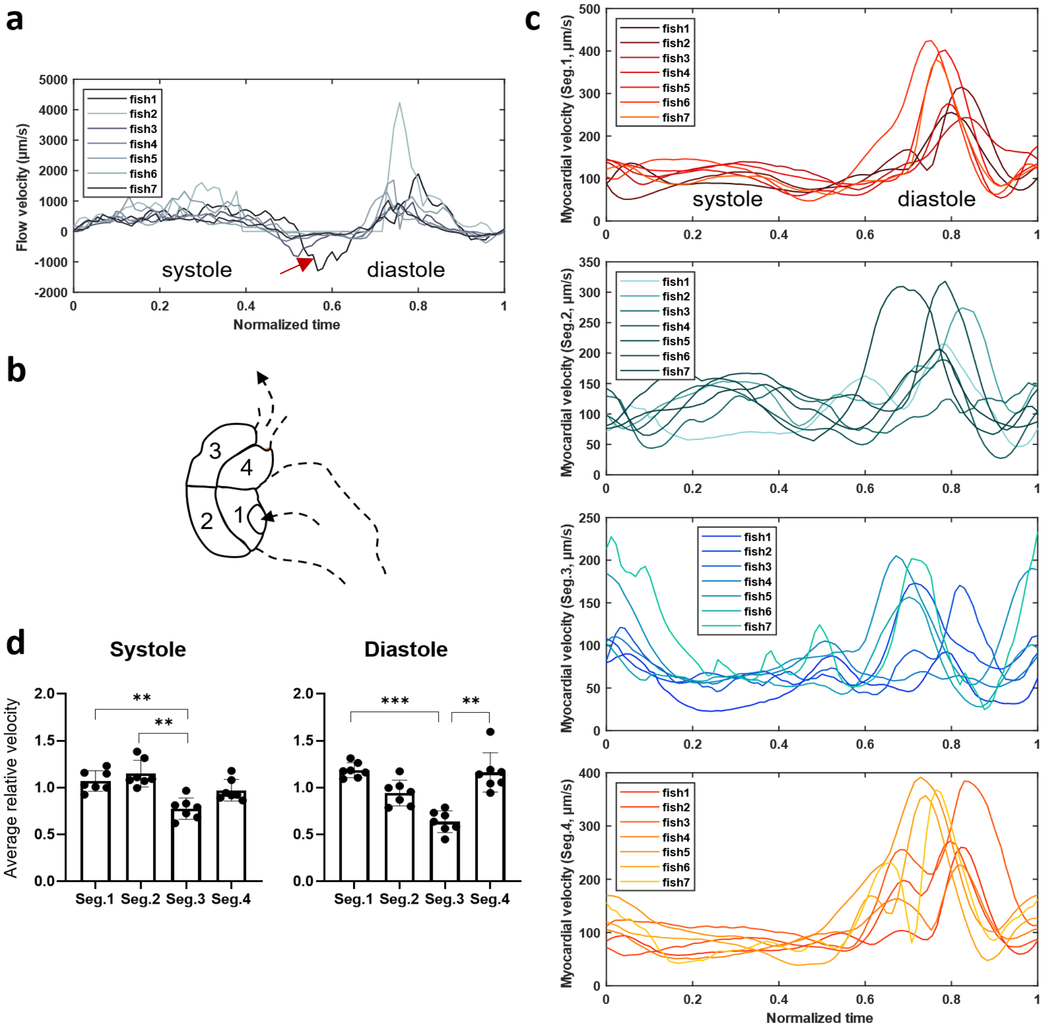

Figure a reveals similar temporal profiles of ventricular flow velocity among fish. The systolic flow appears to be relatively constant and slow, whereas the diastolic flow is instantaneous and rapid. While two fish show significant regurgitant flow (red arrow), the other samples already develop unidirectional flow with only a mild reversal at the end of systole. We considered this as normal phenomenon due to different stages of cardiac development. We expect to obtain more insights into flow reversal from a large dataset and accurate control over the developmental stage of the fish (in hours post fertilization instead of days post fertilization).

For the myocardial displacement, we divided the ventricle into 4 segments in reference to the flow centerline (**figure b**). For each segment, the velocity was tracked in 7 fish and compared among different segments. The results are shown in **figure c**, and we observe the consistent temporal tracing of each segment among the 7 fish. The rapid uprising slope of myocardial displacement velocity during diastole is consistent with the rate of blood flow.

We tried to identify the prominent segment with the strongest displacement in systole and diastole. To minimize the fish-to-fish variation, we normalized the segmental velocity by taking the ratio to the global velocity for each fish heart. The global velocity was defined as the average magnitude of entire ventricular vector map at each time point (manuscript figure 3, lower panel, black dotted line). This ratio was averaged in systole and diastole, respectively. **Figure d** reveals that myocardial contraction (systole) is more prominent in segments 1 and 2 (near the apex) than in segments 3 and 4, whereas myocardial relaxation (diastole) is more prominent in segments 1 and 4 (near the atrium) than in segments 2 and 3.

Overall, the new experimental data strengthened both the shared and variations among the 7 fish at 3 dpf.

3) What is the developmental stage range that this technique can be used in the zebrafish? What are the failure modes that are encountered with respect to resolution, field of view, etc.? It doesn't appear that light penetration is the only limitation given the processing algorithms employed, but an understanding of these limitations would improve the proper use and interpretation of data from the approach.

We imaged the embryonic zebrafish heart from 3 dpf to 5 dpf, and provided the supplementary data to our manuscript (**movie 2**). Factors involved in successful imaging included the heart orientation, heartbeat periodicity (arrhythmia), and blood cell density.

In addition to issues with the accuracy of retrospective gating algorithm, which we have discussed in the response to the Reviewer's Comment #1, the heart orientation will also define the field of view required for the imaging setup, especially in the depth direction. While both light sheet and light field microscopy are limited by light penetration, the latter, in our design, is capable of recovering depth spanning from -76 to 76 μm around the native focal plane. Therefore, positioning the ventricular chamber within this ~150-µm refocus range is needed for an otherwise sub-optimal the light-field reconstruction.

Also, cardiac arrhythmia is not in line with the assumption of the retrospective gating framework. While a small variation (e.g., tens of milliseconds in several frames) is tolerable, our method, in principle, requires that the heart beats periodically.

The blood cell density affects the quality of reconstruction, because light field imaging is well suited to reconstruct the sparse signals. A high density of blood cell distribution leads to the blurred images following reconstruction, and subsequently impedes cell tracking and velocity mapping. We anticipate solving this issue by introducing sparse fluorescence labeling such as heat-shock inducible fluorescent signal (Wagner *et al*., 2019), or by injecting external tracers (Craig *et al*., 2012).
We have included the above paragraph in the Discussion of the manuscript.

Reviewer #2: This article describes a really nice technique to study the couple interaction of myocardial contraction and blood flow dynamics in the zebrafish heart (3 dpf). The technique combines light sheet microscopy and light field microscopy to simultaneously capture myocardial tissue contraction and red blood motion.

Comments and concerns (minor):
1. Deformable image registration, done to capture the myocardium dynamics, may not capture twisting dynamics of the heart tissue. Overall the image registration is best than nothing, but can be misleading too. Please acknowledge this in the discussion.
We appreciate the reviewer's concern. The aim of our analysis is to map the myocardial motion to support following segmental analysis. We expect the algorithm to be compatible with the 3D image data and to be automatic and robust in an unsupervised environment, obviating the human interference.

The commonly used metrics, including ventricular ejection fraction (EF) and fractional shortening (FS), negate the local or segmental myocardial deformation. Both EF and FS rely on human annotation, inevitably entailing inter-observer or user-dependent variation. Alternatively, object tracking is used in echocardiogram for strain analysis, allowing for interrogating a specific region of the myocardium for analysis of myocardial deformation. However, scaling this method to the entire heart, and tracking the myocardium in 3D undergoing continuous deformation remain challenging.

While we agree with the reviewer that image registration does not theoretically correspond to the physical deformation of the myocardium, which can be misleading, we seek to demonstrate the potential of image registration for accurate, robust, and sufficient mapping of the myocardial deformation.

In the following figure, we compared the velocity measured from the manually sampled kymograph with our image registration-based vector map. We used a line across the ventricle to sample the images (**figure (a), scale bar, 30 μm**) over the time domain to generate the kymographs (**figure (b)**). The kymographs visualize the moving boundaries to compute the displacement of the ventricular cavity during cardiac cycles (**figure (c)**, upper row). The derivative of the displacement was calculated as the velocity (**figure c**, middle row), as compared to the one extracted from our vector map (**figure (c)**, lower row; **figure (d)**). The observation indicates that by improperly sampling the heart in a direction (**i**) tilted from the contraction/relaxation direction (**ii**), we dramatically enlarged the displacement measured by kymograph. However, the vector map generated from image registration assessed the myocardial motion in 3D to avoid this sampling error; thereby, providing a reliable measurement in line with the result from the properly sampled kymograph (**figure c, ii**).

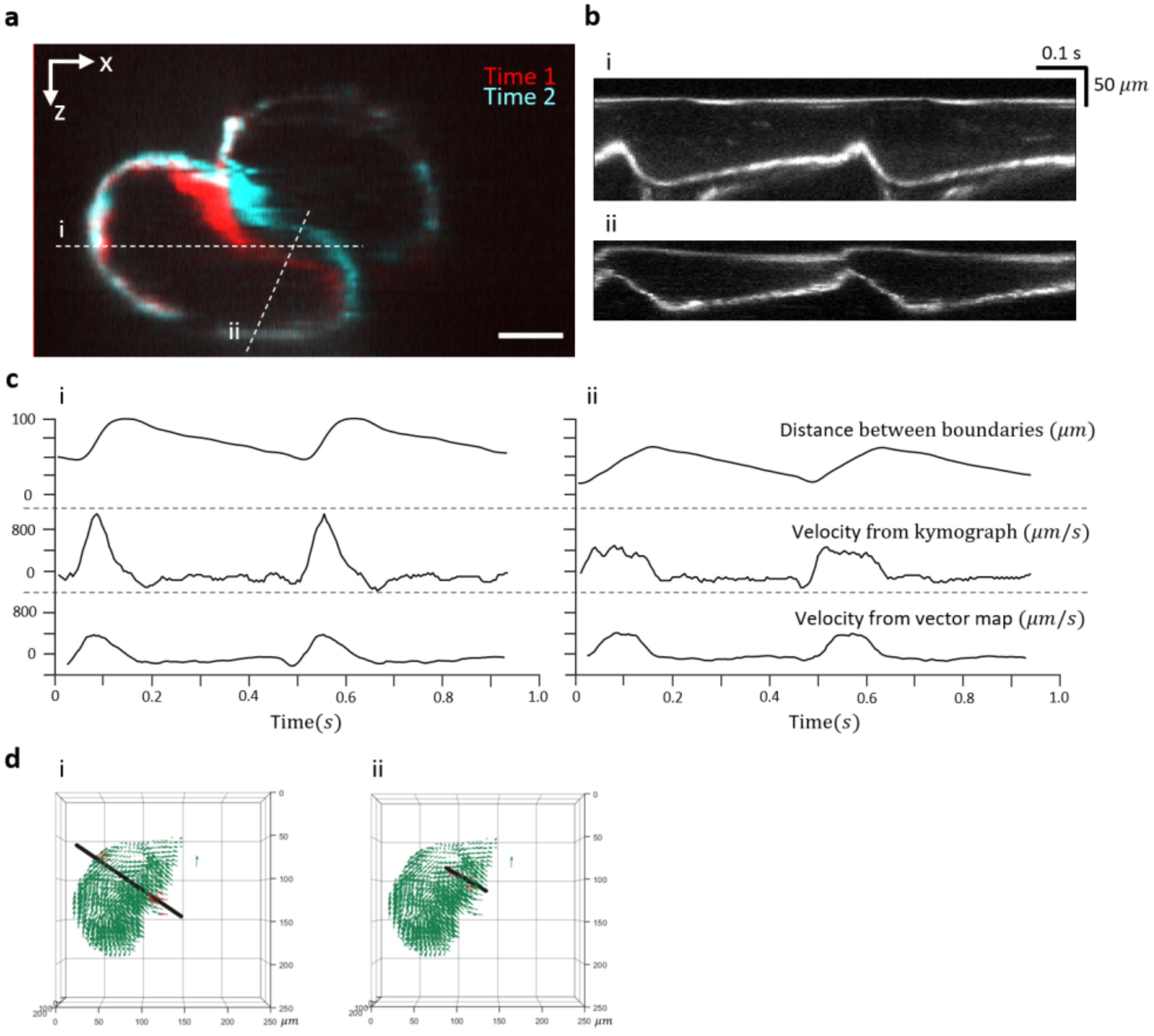

In addition, we further repeated the experiments among other fish under the same condition to demonstrate the consistent observation using the vector mapping (**manuscript figure 4**).

We have added the aforementioned discussion to the revised manuscript (page 13, line 263-274).

2. How was the 'retrospective' synchronization employed checked for accuracy?

The retrospective gating method has been established and validated by Mickoleit *et al.* (2014) and Liebling *et al.* (2005). The accuracy has been addressed in Mickoleit *et al.* (2014), and the reported synchronization error occurs when the “heart contraction wave” propagates nearly along the imaging scanning direction (< 10 degree). In this context, our data (from 3 - 5 days post fertilization) has been collected in the ventral view with the fish’s abdomen facing the objective while the atrium and ventricle are positioned laterally; thus, allowing for maintaining a large angle between the contraction wave and scanning axis. From the reconstructed results, asynchronous displacement between slices at different depths was not observed, indicating sufficient accuracy by retrospective gating method.

3. After reading the description of the blood flow dynamics (results) I had the impression that there was no net flow, given that velocities were negative and negative displacement of red blood cells was about 50% (unless I misunderstood). It is mentioned that the dynamics is inefficient. However, when looking at the movies, the dynamics seem completely different. Blood flow advances and stops, but I cannot see significant backflow. What am I missing? Please describe the dynamics more accurately, or provide better movies.
We have updated **movie 4**, in which we show blood images separately from myocardial images to make the flow motion more obvious. We annotated the flow reversal using red arrows.

4. Are there only very few red blood cells inside the ventricle? Is this accurate?
We thank the reviewer's comment. We have repeated the experiments at 3 dpf to demonstrate the imaging data of the dsRed-labeled blood cells in **movie 4,** in which the density of blood cells varied among different fish. Light-field microscopy has been applied to image the blood cells (Wagner *et al*., 2019; Truong *et al*., 2020; Wang *et al*., 2021). The strength of light-field imaging is to capture the sparse moving signals. Previous studies used heat-shock inducible fluorescence expression (Wagner *et al*., 2019) or *Gata1a* morpholino injection to inhibit hematopoiesis (Wang *et al*., 2021); thereby, reducing the numbers of labeled blood cells. External fluorescent tracer could also be an alternative solution (Craig *et al*., 2012). We expect a better image quality with lower density of blood cells.

5. Comparing the tubular heart of the zebrafish heart analyzed to the mature heart of humans ('apex twisting') is not appropriate, I think. Heart structure is completely different, and while we can learn from the zebrafish, I feel this is not really a similarity to be drawn upon.
We appreciate the reviewer's comments. Torsional contraction in the left ventricle is well-recognized in human cardiac mechanics. In the zebrafish heart, we observed that the direction of myocardial displacement in each segment is distinct but organized. In reference to the reviewer's suggestion, we have modified our statement not to draw a similarity to human heart. To avoid further misunderstanding, we have removed the content related to segmental contractile direction (direction of the displacement) in our manuscript.

Reviewer #3: Wang et al. presented a paper about an image analysis pipeline to integrate light field microscopy (LFM) and light sheet fluorescence microscopy (LSFM) and applied it to blood flow in the heart of developing zebrafish embryos.
Main comment:
It appears hard to exactly capture the achievements that the authors want to highlight in this manuscript. If the author's particular optical integration of LFM and LSFM (i.e. the microscope) has been described in this form for the first time, then the manuscript should highlight that achievement. Currently the microscope is rather taken as a given (author's reference 20-22) and described in detail elsewhere (author's reference 22).

Similarly, the postprocessing of LFM images is complicated but seems to have been published and highlighted elsewhere (author's reference 22). The reviewer acknowledges that author's reference #22 is a preprint, and that the current manuscript's Fig 1 almost identically appears as Fig 3 in the 2019 bioRxiv version of #22. But the 2020 version of #22 uses different figures and is clearly focused on microscopy and deep learning data processing. So the reviewer assumes that microscopy and deep learning are not part of the current manuscript under review.
If we assume that the paper starts with LFM and LSFM images and aims to highlight the achievement of creating a data processing pipeline, then it is similarly hard to exactly pinpoint the author's scientific achievement highlighted in the manuscript.
It seems that the deformable image registration (DIR) used to perform vector analysis stems from the standard MATLAB toolbox and similarly, it seems that the particle tracking velocimetry (PTV) analysis is done using standard Imaris functions (see Methods 'Computation of Vector Mappings'). The authors refer to a github repository https://github.com/aaronzq/cardiac but at the time of this review, the reviewer received an '404 webpage not available' error (screenshot below). Similarly, looking through aaronzq's 18 public github repositories, no 'cardiac' repository could be found. Aaronzq's vcdlfm repo comes with a nice jupyter notebook tutorial but seems to be unrelated to this manuscript. So unfortunately, while code in the github repository could have clarified some of the questions above as to what the author's contributions to the field are, it was unfortunately not available for this review.
From personal experience, the reviewer knows how hard it is to create an end-to-end imaging system plus image processing pipeline. If this is what the authors did, and in addition creating a working application on the developing zebrafish heart, then that is fantastic. However the way the manuscript is written, it is unclear what the main achievement is and what should be highlighted.
In general, the manuscript would benefit from a clear purpose:
E.g. if:
Computational question:
- No comparison between an old tool and a new tool (PIV vs PTV is indicated in discussion but just one sentence)
Engineering question:
- Is it hard to do LFSM and LFM together? What is the prior art here?
Biological question:
- No comparison to mutants (disease)
- No comparison to different times dpf (development)
Combined Engineering, computational and biological question:
- What is the prior art? Could this never be imaged before?
- And if so, what are the surprising insights you learned, now that you see it?
In general, the manuscript would benefit a lot from a deeper description of the prior art (literature review), the aim to improve on this prior art, the obstacles along the way of implementing these improvements and the eventual result from that effort.
The reviewer thinks that the manuscript has a lot of potential. Combining LSFM and LFM is fantastic (but is it the aim of this paper?). The deep learning to process LFM is important (is it the aim of this paper?). The generation of a data processing pipeline to go from raw zebrafish heart LSFM and LFM imaging data to processed insights is

important (is that the aim of this paper? How much of the work is from the authors vs the use of standard library functions of MATLAB and Imaris? An accessible github repository would help to clarify that question). The insight gained from imaging zebrafish hearts at that speed volumetrically is intriguing! (is that the aim of the paper? Where is the comparison to prior art, what is the new data? What are the surprising findings?). And if the aim is more biological: Where is the comparison to other developmental timepoints? Does the bloodflow heart dynamics change? How does the data change when mutations or other altered conditions are applied?
The reviewer recommends 1) a major revision and 2) recommends that the authors rewrite the manuscript to clarify the above questions and 3) make their github repo available to support their claims.

We appreciate the reviewer’s comments. We have performed a major revision of the introduction and discussion sections.

As a summary, we intended to highlight the imaging pipeline (a hybrid hardware enabled by a modified retrospective gating algorithm). This pipeline enables the synchronized visualization of both the beating myocardium and intracardiac flowing dynamics in the embryonic zebrafish hearts. We hereby clarify that following key points:

1. Combining LSFM and LFM for embryonic zebrafish heart represents the conceptual innovation and novel imaging modality in this work. We customized and implemented the retrospective gating method to synchronize the image data from two modalities. A hybrid imaging setup was built to support the synchronization algorithms. The data generated by this method is demonstrated to enable motion analysis of the cardiac dynamics.
2. Using deep learning to process LFM data is based on our recently reported work (Wang *et al.*, 2021).
3. We developed the image processing pipeline used to generate velocity analysis from raw image data. However, the particle tracking and image registration rely on the standard library functions of MATLAB and Imaris.
4. The GitHub repository should be accessible now.
5. We have not included mutations nor pharmacological manipulations in the current work; however, our laboratories have experience and expertise in applying *Gata1a* or *Tnnt2a* morpholinos for loss-of-function and EPO mRNA for gain-of-function to study shear stress-mediated cardiac trabeculation and outflow tract valvulogenesis (Lee *et al*., 2016, Lee *et al.,* 2018, Hsu *et al*., 2019).

Minor comments:
- The authors talk about 200 fps and 200 vps. That might be confusing for some readers. The exact difference should be stated.
200 fps: 200 2D frames per second
200 vps: 200 3D volumes per second, a volume consists of a stack of 2D slices

The following clarification is added to the manuscript (page 5, line 84-85).
(vps, defined as the number of acquired stacks of 2-D cross-sections of the sample per second)

- Similarly: The exact difference between LFM and LSFM needs clarification. Two example sentences from the author's manuscript: "The LSFM system was capable of rapidly visualizing the embryonic zebrafish heart at 200 frames per second", "For this reason, light-field requires merely a single exposure for fast volumetric (3-D) detection up to 200 frames per seconds [23]" So are they the same speed? Why put them together then?

Light field has the capacity to reconstruct 3D image from the 2D frame, and the 200 frames per second in LFM represent 200 volumes per second after reconstruction. However, LSFM usually requires scanning across the sample to obtain a 3D stack. Multiple frames need to be acquired for reconstructing into a volume. Retrospective gating brings a new approach for LSFM to image the volume containing periodic signals. LSFM acquires a series of 2D image sequences at each scanning step, followed by synchronizing these sequences to reconstruct 3D volumes. In this case, the 3D imaging speed is identical to the 2D frame rate at 200 frames per second (when capturing each 2D sequence). Therefore, we used LSFM for high resolution imaging of periodic myocardial motion and LFM for instantaneous imaging of random but sparse flowing blood cells.

- Similarly, the author's sentence "Owing to the limited 3-D imaging speed associated with the current optical microscopy, uncoupling myocardial contraction from intracardiac flow dynamics to investigate ventricular structure and function remains an imaging challenge" does not specify what these limitations are (missing prior art review) and why specifically potentially combining two modalities could solve those challenges.

We have revised the introduction section to include the prior art review. Briefly, LSFM with retrospective gating method has successfully captured the contracting myocardium. However, LSFM is limited from acquiring the flowing blood cells. This random motion of flow blood cells is not in line with the periodicity assumption in retrospective gating (Mickoleit *et al.*, 2014; Liebling *et al.,* 2005). To address this biomedical imaging challenge, we have introduced the LFM for its capacity to instantaneously acquire the 3D imaging of sparse signals such as the blood cells. In LFM, volumetric detection of moving blood cells does not rely on the periodic property of motion and can be synchronized with LSFM data using our hybrid imaging pipeline. Therefore, this combination of LFM and LSFM allows for the volumetric detection of intracardiac flow dynamics with the contracting myocardium.

- Fig. 2D, the range 'low to high' would benefit from having physical units

We have added the physical units.

Typos:

- The manuscript would benefit from a spell checker-run before re-submission - "DIR with PTV techqniues "
- "uncovers sptatial"
- "flow dynamics to study to cardiac morphogenesis"

We have rectified the typos in the revised manuscript.

References:

Mickoleit, M., et al. (2014). "High-resolution reconstruction of the beating zebrafish heart." Nat Methods **11**(9): 919-922.

Liebling, M., et al. (2005). "Four-dimensional cardiac imaging in living embryos via postacquisition synchronization of nongated slice sequences." J Biomed Opt **10**(5): 054001.

Lee, J., et al. (2016). "4-Dimensional light-sheet microscopy to elucidate shear stress modulation of cardiac trabeculation." The Journal of Clinical Investigation **126**(5): 1679-1690.

Vedula, V., et al. (2017). "A method to quantify mechanobiologic forces during zebrafish cardiac development using 4-D light sheet imaging and computational modeling." PLoS Comput Biol **13**(10): e1005828.

Weber, M., et al. (2017). "Cell-accurate optical mapping across the entire developing heart." eLife **6**: e28307.

Wagner, N., et al. (2019). "Instantaneous isotropic volumetric imaging of fast biological processes." Nat Methods **16**(6): 497-500.

Truong, T. V., et al. (2020). "High-contrast, synchronous volumetric imaging with selective volume illumination microscopy." Communications Biology **3**(1): 74.

Wang, Z., et al. (2021). "Real-time volumetric reconstruction of biological dynamics with light-field microscopy and deep learning." Nat Methods.

Craig, M. P., et al. (2012). "An optimized method for delivering flow tracer particles to intravital fluid environments in the developing zebrafish." Zebrafish **9**(3): 108-119.

Lee, J., et al. (2018). "Spatial and temporal variations in hemodynamic forces initiate cardiac trabeculation." JCI insight 3.13.

Hsu, J. J. et al. (2019). “Contractile and hemodynamic forces coordinate Notch1b-mediated outflow tract valve formation.” JCI insight vol. 5,10 e124460.
