# Supplementary material for: A hybrid of light-field and light-sheet imaging to study myocardial function and intracardiac blood flow during zebrafish development
Source: PLoS Comput Biol. 2021 Jul 6;17(7):e1009175. doi: 10.1371/journal.pcbi.1009175 (PMC8284633; doi:10.1371/journal.pcbi.1009175)
Supplement: Attachment — Submitted filename: response_letter_05112021.pdf [file pcbi.1009175.s014.pdf]

Reviewer #1: Overall the authors have been responsive to all of the reviewers comments, in particular with the generation of additional data with respect to multiple embryos to elaborate spatial/temporal variation in fluid and myocardial data. In their response to reviewers they acknowledge the challenge in scaling 3D/4D deformation (tissue strain) maps of the embryonic heart, but it is unclear whether those maps are indeed possible to be generated with the technology the authors have developed. It is unclear if their computational approach is capable of rendering fluid or solid biomechanical data as the title indicates. Fluid and tissue velocity profiles are interesting, but not the same as the functionally relevant tissue wall strain and shear stress. These parameters require reference frame decomposition and relative motion measurements between data points in the cloud, while single point velocity tracts (or averaging from a whole segment) does not. It is unclear to what degree of this biomechanically relevant data the authors claim their system is capable to do using the velocity data they currently have. If they indeed have point-to-point information of their solid boundaries and/or complete grids of fluid velocity data (as suggested in their vector maps), it should be straightforward to do. If not, how are their datasets digitized for analysis?

We agree with the reviewer's insightful comment. We hereby humbly clarify the degree to which our data may address biomechanically relevant zebrafish model.

1. Strain and strain rate

We computed the displacement and velocity of the ventricular wall motion during a cardiac cycle. Due to regional/segmental variations in the ventricular contraction, we adopted the method of strain and strain rate to quantify the deformation of the ventricular wall as follows (Dandel M, *et al.*, 2009):

$$Strain: \varepsilon = \frac{L-L_0}{L_0} = \frac{\Delta L}{L_0}, \qquad \text{equation (1)}$$

$$Strain\ rate: \dot{\varepsilon} = \frac{d\varepsilon}{dt} = \frac{d(\Delta L/L_0)}{dt} = \frac{\Delta V}{L_0}, \qquad \text{equation (2)}$$

where L_0 and L denote the baseline and instantaneous displacement of the specific region/segment of the ventricle, and dV refers to the change in velocity, or velocity gradient. Our velocity map supports the velocity gradient for calculating the strain rate from equation (2), as demonstrated in Figure 1. The strain can be further computed by integrating the strain rate.

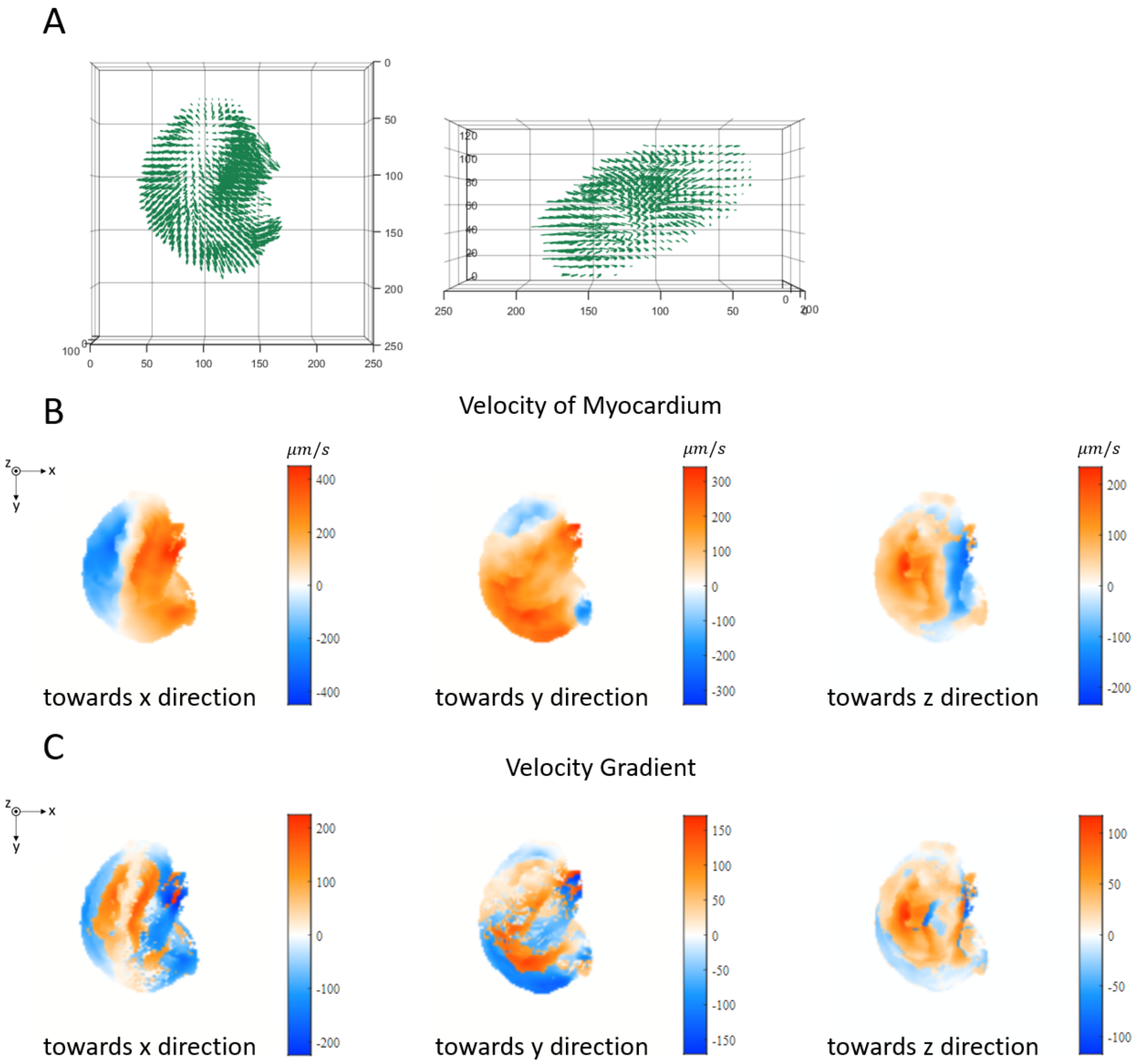

Figure 1. (**A**) The velocity field illustrates ventricular contraction from the superior and lateral views of the ventricle. (**B**) The velocity of ventricular contraction is depicted in the x, y and z axis. (C) The gradient of velocity in the x, y and z axis reveals the differential rates of regional/segmental wall deformation.

The deformable image registration-based method has been reported by other investigators to analyze strain (Christen, *et al.*, 2012; Heyde, *et al.*, 2012; Liu, *et al.*, 2020; Yang, *et al.*, 2017). In line with this method, our light-sheet imaging system and processing pipeline generate displacement/velocity map, allowing for the time-dependent regional strain analysis of the ventricle.

In some studies, investigators have adopted the generalized definition of strain (equation 1) by measuring the changes in ventricular diameters as the L_0 and L during cardiac cycle (Lee, *et al.*, 2016; Hsu, *et al.*, 2019; Chen, *et al.*, 2019). The rate of changing diameters, similar to strain rate, is used to reflect ventricular deformation. In Figure 2 below, we demonstrated how we use

our dataset to compute the rate of ventricular cavity deformation: the region of interest (ROI) was defined by the intersection between our sampling line (i or ii) and the ventricular walls. We sampled the velocity map in the ROI and computed the average velocity of the wall motion at that site. By taking the difference between the velocity on the opposite walls, we were able to derive the velocity of deformation.

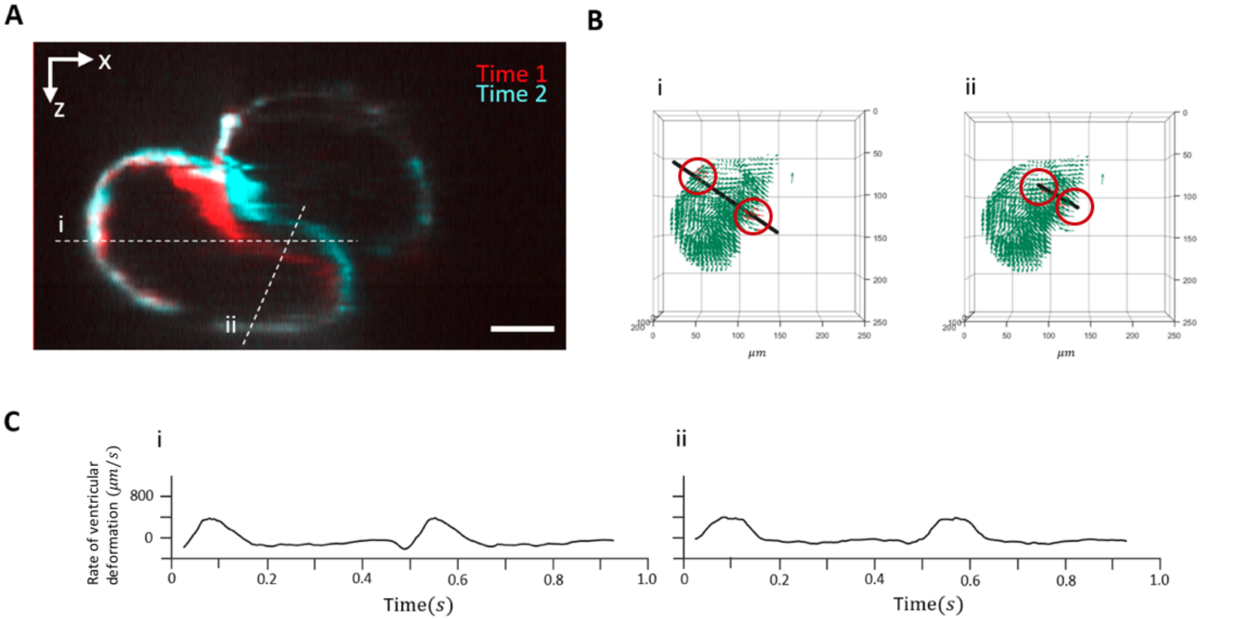

Figure 2. (**A**) Two dotted lines (i and ii) cross the x-z plane of the ventricle at two different time points (Time 1 vs. Time 2). (**B**) The velocities are sampled at the intersections of the line (i or ii) and the ventricular wall. (**C**) The rates of ventricular deformation are derived from the velocity difference at the two opposite intersection points (i or ii).

2. Shear stress

The wall shear stress (WSS) at steady state is defined as:

$$WSS = \mu \frac{\partial v}{\partial y}, \qquad \text{equation (3)}$$

where μ is the viscosity, dv/dy is the velocity gradient in the normal direction to the wall. Accurate computation of the velocity gradient requires a high-resolution velocity map near the wall. In our case, light-field imaging was used to detect the trajectories of individual blood cells traveling from atrium to ventricle, and the sparse velocity mapping was a limitation to acquire velocity gradient near the wall needed for accurate calculation of endocardial shear stress.

In this context, we agree with the reviewer's concern and decide to modify the title of our manuscript.

Reviewer #2: Concerns from reviewers 1 & 2 have mainly not been addressed. There is a response - but frequently not to the point and not directly addressing the reviewers concerns or aspects for clarification. As an example, the question of whether the retrospective synchronization was validated (reviewer 2, issue #2) is not addressed. I am guessing the answer is no. To validate you need to acquire images at different angles - including an angle that shows the wave propagation - the retrospective synchronization usually needs to be 'adjusted' with that information. A similar question, I believe, is asked by reviewer 1, (#1 and #2) and also not directly addressed.

Overall, however, answers to reviewer #3 seem to have been addressed. In particular the introduction and discussion, which have been completely re-written, seem to be much more focus on the actual contribution of the paper. These sections are now a really nice description of the methods employed and what is new in this paper - the combination of LSM and LSFM - to both capture myocardium motion and red blood cell velocities.

We thank the reviewer's comments. In reference to the previously published work by Mickoleit et al., 2014; Liebling et al., 2005 and successful applications (Lee et al., 2016; Vedula et al., 2017; Weber et al., 2017), we did not validate our data for the retrospective synchronization. The published validation focused on the pitfalls for using synchronization to address the "heart contraction wave" propagation from the atrium to ventricle along the imaging scanning direction. Therefore, we avoided this case and adopted the ventral view in our zebrafish imaging.

Previous reviewer 1:
#2 "it is unclear how the local features measured in either tissue kinematics/velocities are shared in embryos of the same stage or across stages": We have obtained a large dataset featuring 7 fish

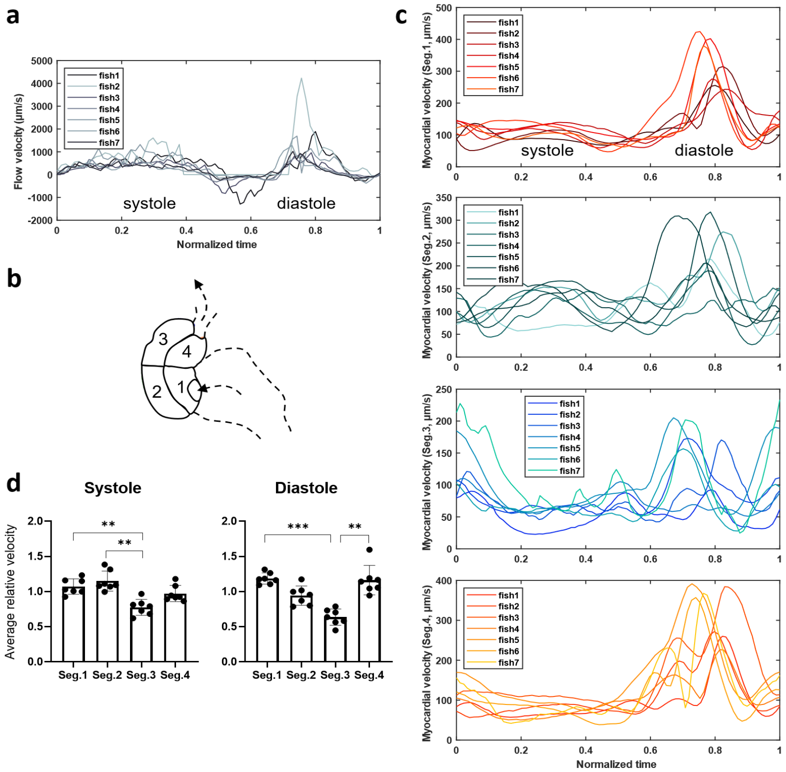

at the same developmental stage.
#3 “What is the developmental stage range that this technique can be used in the zebrafish? ”: We presented the dataset from 3 to 5 days post fertilization (dpf).
“What are the failure modes that are encountered with respect to resolution, field of view, etc.? ”: We discussed the failure mode in terms of zebrafish heart orientation, field of view (depth direction), ventricular arrhythmia, and blood cell density/viscosity.

Previous reviewer 2:
#1 “Deformable image registration, done to capture the myocardium dynamics, may not capture twisting dynamics of the heart tissue. Overall the image registration is best than nothing, but can be misleading too. Please acknowledge this in the discussion.

We agree with the reviewer, and we acknowledged this limitation in our Discussion. However, compared to the manual measurement (by image kymograph), the image registration-based measurement demonstrates the efficacy in quantifying displacement/velocity and is less sensitive to angles.

#2 This comment is similar to that of Reviewer 1 #1. To validate the accuracy, we refer to previously published work (Mickoleit *et al*., 2014; Liebling *et al*., 2005; Lee *et al.,* 2016; Vedula *et al*., 2017; Weber *et al*., 2017).

#3 The reviewer pointed out that the backflow (regurgitation) in our video is not as visible as compared to our quantification. For this reason, we have annotated the movie.

#4 “Are there only very few red blood cells inside the ventricle? Is this accurate?”: The density of the blood cells varied among different fish at different stages of the development (please refer to the videos). We cited the publications in which the identical technique was used to image the blood cells (Wagner *et al*., 2019; Truong *et al*., 2020; Wang *et al*., 2021).

#5 “Comparing the tubular heart of the zebrafish heart analyzed to the mature heart of humans ('apex twisting') is not appropriate, I think. ”: We agree with the reviewer’s suggestion, and we removed “segmental contractile direction”.

References:

Dandel M, Lehmkuhl H, Knosalla C, Suramelashvili N, Hetzer R. Strain and strain rate imaging by echocardiography - basic concepts and clinical applicability. Curr Cardiol Rev. 2009;5(2):133-148.

Christen, D., Levchuk, A., Schori, S., Schneider, P., Boyd, S.K. and Müller, R., 2012. Deformable image registration and 3D strain mapping for the quantitative assessment of cortical bone microdamage. Journal of the mechanical behavior of biomedical materials, 8, pp.184-193.

Heyde, B., Jasaityte, R., Barbosa, D., Robesyn, V., Bouchez, S., Wouters, P., Maes, F., Claus, P. and D'hooge, J., 2012. Elastic image registration versus speckle tracking for 2-d myocardial motion estimation: A direct comparison in vivo. IEEE transactions on medical imaging, 32(2), pp.449-459.

Liu, J., Li, Y., Cui, Y., Cao, Y., Yao, S., Zhou, X., Wetzl, J., Zeng, W. and Shi, H., 2020. Quantification of myocardial strain in patients with isolated left ventricular non-compaction and healthy subjects using deformable registration algorithm: comparison with feature tracking. BMC Cardiovascular Disorders, 20(1), pp.1-9.

Yang, X., Torres, M., Kirkpatrick, S., Curran, W.J. and Liu, T., 2017. Ultrasound 2D strain measurement for arm lymphedema using deformable registration: A feasibility study. PloS one, 12(8), p.e0181250.

Lee, J., et al. (2016). "4-Dimensional light-sheet microscopy to elucidate shear stress modulation of cardiac trabeculation." The Journal of Clinical Investigation 126(5): 1679-1690.

Hsu, Jeffrey J., et al. "Contractile and hemodynamic forces coordinate Notch1b-mediated outflow tract valve formation." JCI insight 4.10 (2019).

Chen, Junjie, et al. "Displacement analysis of myocardial mechanical deformation (DIAMOND) reveals segmental susceptibility to doxorubicin-induced injury and regeneration." JCI insight 4.8 (2019).

Mickoleit, M., et al. (2014). "High-resolution reconstruction of the beating zebrafish heart." Nat Methods 11(9): 919-922.

Liebling, M., et al. (2005). "Four-dimensional cardiac imaging in living embryos via postacquisition synchronization of nongated slice sequences." J Biomed Opt 10(5): 054001.

Vedula, V., et al. (2017). "A method to quantify mechanobiologic forces during zebrafish cardiac development using 4-D light sheet imaging and computational modeling." PLoS Comput Biol 13(10): e1005828.

Weber, M., et al. (2017). "Cell-accurate optical mapping across the entire developing heart." eLife 6: e28307.

Wagner, N., et al. (2019). "Instantaneous isotropic volumetric imaging of fast biological processes." Nat Methods **16**(6): 497-500.

Truong, T. V., et al. (2020). "High-contrast, synchronous volumetric imaging with selective volume illumination microscopy." Communications Biology **3**(1): 74.

Wang, Z., et al. (2021). "Real-time volumetric reconstruction of biological dynamics with light-field microscopy and deep learning." Nat Methods.
